# Unconventional picosecond strain pulses resulting from the saturation of magnetic stress within a photoexcited rare earth layer

**DOI:** 10.1063/1.5145315

**Published:** 2020-03-27

**Authors:** A. von Reppert, M. Mattern, J.-E. Pudell, S. P. Zeuschner, K. Dumesnil, M. Bargheer

**Affiliations:** 1 Institut für Physik & Astronomie, Universität Potsdam, Karl-Liebknecht-Str. 24-25, 14476 Potsdam, Germany; 2 Helmholtz Zentrum Berlin, Albert-Einstein-Str. 15, 12489 Berlin, Germany; 3 Institut Jean Lamour (UMR CNRS 7198), Université Lorraine, 54000 Nancy, France

## Abstract

Optical excitation of spin-ordered rare earth metals triggers a complex response of the crystal lattice since expansive stresses from electron and phonon excitations compete with a contractive stress induced by spin disorder. Using ultrafast x-ray diffraction experiments, we study the layer specific strain response of a dysprosium film within a metallic heterostructure upon femtosecond laser-excitation. The elastic and diffusive transport of energy to an adjacent, non-excited detection layer clearly separates the contributions of strain pulses and thermal excitations in the time domain. We find that energy transfer processes to magnetic excitations significantly modify the observed conventional bipolar strain wave into a unipolar pulse. By modeling the spin system as a saturable energy reservoir that generates substantial contractive stress on ultrafast timescales, we can reproduce the observed strain response and estimate the time- and space dependent magnetic stress. The saturation of the magnetic stress contribution yields a non-monotonous total stress within the nanolayer, which leads to unconventional picosecond strain pulses.

## I. INTRODUCTION

Experiments that probe the strain response of the atomic lattice that results from the light-matter interaction of a femtosecond optical pulse with an opto-acoustic transducer material can be subsumed as picosecond ultrasonics.^1,2^ They yield fundamental insights into physical processes within the laser-excited thin film, such as electron-phonon coupling,^3–5^ hot electron propagation,^6,7^ and electron–hole pair generation.^8,9^ This is possible because the lattice strain is the deterministic, elastic response to a physical stress that itself contains the time- and length-scales of the energy transfer processes within the transducer region. Research in this field has developed from studying the elementary processes in metals^3,4^ and semiconductors^8,10^ to the point that various thermal and non-thermal mechanisms for the stress generation have been distinguished.^11^

Picosecond ultrasonics within magnetic materials offers a route to study spin-lattice interactions in the time domain. An additional motivation comes from the prospect that strain assisted magnetization manipulation could lead to faster, potentially field-free data storage techniques, with increased storage densities.^12,13^ Recent experiments have shown that electronically generated surface-acoustic-waves are able to switch the magnetization by nanosecond strain pulses.^14,15^ Precession of the magnetization due to traversing picosecond strain pulses that transiently modify the crystal field anisotropy has been observed in many common and technologically relevant magnets such as nickel,^16,17^ GaMnAs,^18,19^ galfenol,^20^ and doped yttrium–iron–garnet.^21,22^ The inverse effect, i.e., lattice stress that originates from the change of the magnetic state, is less explored by time-resolved investigations although examples, such as the metamagnetic phase-transition in FeRh^23,24^ and the change in the tetragonality of FePt^25,26^ and SrRuO_3_,^27^ exist. For static and low frequency applications, it is known that metallic, magnetostrictive transducers complement the frequently used piezoelectric ceramics with the advantage of increased conductivity and ductility.^28^ The class of heavy rare earth elements exhibits an exceptionally large magnetostriction^29^ where the stress that can be generated by spin disorder is not only contractive but also dominates over the expansive phonon contribution as we have confirmed by probing the structural response of laser-excited gadolinium,^30^ holmium,^31^ and dysprosium (Dy).^32,33^ Ultrafast x-ray diffraction (UXRD) is a suitable tool for quantitatively probing the strain generation and propagation as well as the accompanying heat flow in crystalline heterostructures that are either inseparable or potentially opaque to all-optical probing schemes.^34–36^

Here, we present the ultrafast lattice response of a laser-excited Dy thin film within a metallic heterostructure, where we use a buried niobium (Nb) layer for separating strain pulses from the lattice expansion that results from heat diffusion. We systematically analyze the lattice dynamics as a function of the temperature-dependent magnetic order and the laser excitation energy density. The strain-pulse observed in the Nb detection layer changes upon cooling well below $T_{Né\text{el}}$ from a bipolar compression-expansion feature that is characteristic of a fast expansive stress to an almost unipolar expansion that results from a slowly rising contractive stress within the transducer. By modeling the strain response of the heterostructure, we obtain separated spatio-temporal stress-profiles for both the expansive phonon stress and the contractive magnetic stress. The stress within the Dy layer changes from expansive to contractive because the energy conversion to the spin system is saturated only in the strongly excited near surface region. To reproduce the observed low-temperature strain-response within a one dimensional elastic model, we have to assume a contractive stress contribution that rises nearly instantaneously and counteracts the quasi-instantaneous expansive, thermoelastic stress from hot phonons and electrons. In addition, a second contractive contribution is needed that rises with an ≈15 ps time constant. These timescales match the sub-picosecond electron-spin coupling and the subsequent phonon-spin coupling that were reported by previous demagnetization experiments in heavy rare earth elements.^37–39^ For high excitation densities, we observe an additional increase in the spin-stress on a longer timescale. We attribute this to phonon mediated energy transport processes from surface-near regions of complete demagnetization to regions with partial magnetic order deeper in the sample, which have been photoexcited less. The energy transfer to the magnetic system removes energy from the phonon system. The storage of heat in spin disorder in Dy is documented by a reduced thermal expansion of the buried Nb detection layer, which only accepts heat from electrons and phonons.

The presented experiments extend our previous works^32,33^ that mainly discuss the evolution of the average Dy layer strain on timescales *t *>* *45 ps, by an analysis of the initial picosecond strain response for *t *<* *180 ps. The main experimental novelty is the use of a dedicated non-magnetic and non-excited detection layer that allows for a clear separation of elastic waves and thermal expansion following the diffusion of heat. In addition, we now discuss a spatially resolved model for the magnetic stress evolution and its saturation within the Dy layer, which is at the origin of the unconventional picosecond strain response.

This paper consists of three main parts: in Sec. II, we present the sample characterization and the temperature dependent lattice expansion of the transducer and detection layer and introduce the concept of Grüneisen constants that are central to the following analysis. Section III contains the main experimental findings on the temperature and excitation energy dependent strain within the transducer and detection layer studied by UXRD. Section IV is devoted to the modeling of the spatiotemporal stress profile and corroborates the experimental findings.

## II. STATIC PROPERTIES

In the following, we provide a brief overview over the relevant properties that are later probed by time-resolved experiments. We depict a representative x-ray diffraction pattern and discuss the material specific thermal expansion response that shows fingerprints of the magnetic phase-transition within the Dy layer. We introduce the thermodynamic concept of a Grüneisen constant that relates the energy density for phonons and magnetic excitations to their stress on the crystal lattice.

### A. Sample characterization by x-ray diffraction

**FIG. 1.**
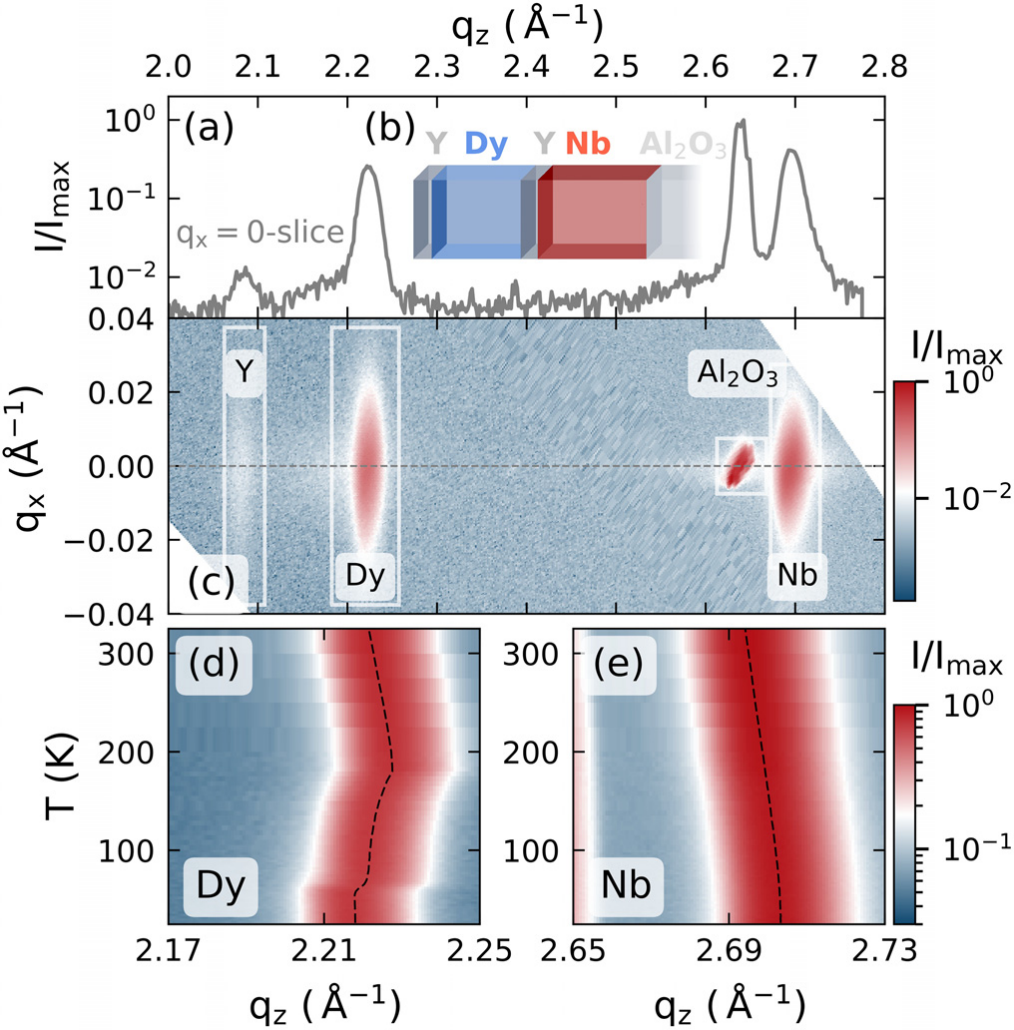
Static x-ray diffraction results: (a) x-ray diffraction intensity of the sample structure that is schematically depicted in the inset (b). The material specific Bragg peaks are labeled in the reciprocal space map shown in (c). The temperature dependent peak-shifts that contrast the negative thermal expansion in the FM and AFM phase of dysprosium to the monotonous peak shift of the PM niobium are depicted in (d) and (e), respectively.

The static and temperature dependent characterization of the Dy sample by x-ray diffraction is provided in Fig. 1. In the inset of Fig. 1(b), we display a schematic of the investigated metallic heterostructure. It consists of a 80 nm (0001)-oriented Dy transducer layer grown in between two yttrium (Y) hcp-(0001) layers (22 nm on top of and 5 nm below Dy) on-top of a 102 nm niobium (Nb) body centered cubic (110)-oriented film that enables the crystalline growth on a sapphire (Al_2_O_3_) hcp-(11–20) substrate^40^ and serves as an additional strain detection layer. X-ray diffraction, using a microfocus ${\text{Cu}-K}_{\alpha }$ radiation source, yields a reciprocal space projection that is depicted in Fig. 1(c), where the four different diffraction maxima that correspond to the material specific lattice constants are clearly separated along the out-of-plane reciprocal-space coordinate *q_z_*. These Bragg-peaks are seen as diffraction intensity maxima in Fig. 1(a), where we show the slice of the reciprocal space at *q_x_* = 0. In the main experiments, we observe the time-dependent shift of the Dy and Nb diffraction peaks after laser-excitation and extract the resulting average lattice strain *η* of these materials in a laser-pump, x-ray diffraction-probe scheme, with a time-resolution of approximately 200 fs as described previously.^35,41,42^

An important reference for our interpretation of the lattice response upon laser-excitation is the equilibrium thermal expansion and contraction during heating of the sample structure. The temperature dependent peak-shift that we extract by heating from 25 K to 350 K is depicted in Figs. 1(d) and 1(e) for Dy and Nb, respectively, where the dashed lines indicate the peak center positions as obtained by Gaussian fits. The monotonous shift of the niobium peak to smaller *q_z_* represents the common positive thermal expansion behavior. This contrasts with the thermal expansion seen in the dysprosium peak that exhibits a pronounced negative thermal expansion (NTE) between 40 K and 180 K as well as a change between expansion and contraction at 180 K.

### B. Temperature-dependent material properties

**FIG. 2.**
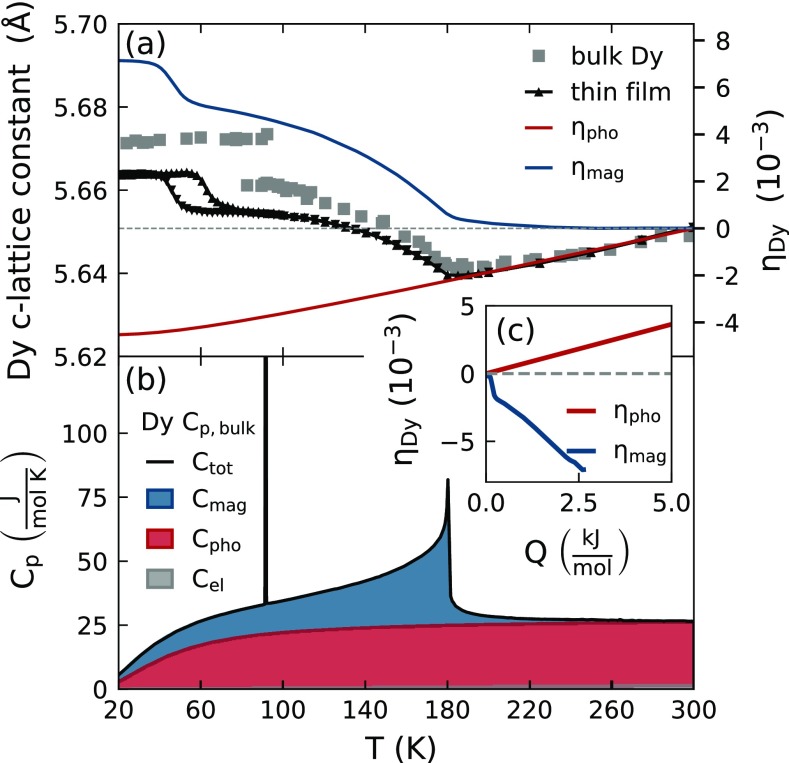
Subsystem separation of (a) the static strain and (b) heat capacity contributions in Dy: The temperature-dependent *c*-axis of Dy and the heat capacity show a pronounced change at the AFM-PM phase transition at $T_{Né\text{el}}\approx 180$ K. The FM-AFM phase transition that occurs at ${\text{T}}_{\text{Curie}}\approx 90$ K for bulk Dy is shifted to lower temperatures in the used thin film sample. The inset in (c) shows the strain per deposited energy, which results from the separation of the strain and heat capacity into the contributions of phonon and spin excitations, which are indicated in red and blue, respectively.

The temperature dependent lattice strain $\eta =(c(T)-c_{0})/c_{0})$ of the *c*-axis with $c_{0}=c(T=250\;K)$ in the hcp unit cell of the investigated thin Dy film is depicted in Fig. 2. For comparison, we relate it to the lattice constant change^43^ [Fig. 2(a)] and to the heat capacity^44^ of bulk Dy [Fig. 2(b)]. Changes in the thermal expansion are known to coincide with changes within the magnetic order and magnetic contributions to the strain can exceed 10^−3^.^29,45^ Elemental Dy has one of the highest magnetic moments of $10.64\;\mu_{B}$ per atom, which order ferromagnetically (FM) below $T_{\text{Curie},\text{bulk}}\approx 90$ K and antiferromagnetically (AFM) between $T_{\text{Curie}}$ and $T_{Né\text{el}}\approx 180$ K above which Dy becomes paramagnetic (PM).^46–48^ The large magnetic moment originates mainly from the localized magnetic moments of the partially filled 4*f*-electron orbitals that interact via the delocalized 5d6s-conduction band electrons by the Ruderman -Kittel -Kasuya -Yosida (RKKY)-mechanism.^47,48^ The magnetic easy axis in the FM phase lies along the *a*-axis in the basal plane of the hexagonal unit cell and the AFM phase exhibits a helical spin ordering that is characterized by a finite turn angle between the magnetic moments for neighboring unit cells along the *c*-axis direction.^46^

Magnetostriction in rare earth elements is often discussed within the so-called standard model of magnetostriction pioneered by Callen and Callen.^49,50^ This formalism takes both single-ion and two-ion interactions into account.^50,51^ Single-ion contributions originate from the interaction of the crystal field with the anisotropic 4*f*-orbitals, which leads to a lattice deformation upon magnetization change due to the intrinsic spin–orbit coupling.^51,52^ The case of vanishing orbital momentum, that is realized in Gadolinium, demonstrates the importance of the exchange-striction mechanism that explains the occurrence of magnetostriction even for spherically symmetric charge distributions.^51^ Exchange-striction is a two-ion contribution that originates from a distance-dependent magnetic interaction energy (here provided by the oscillatory RKKY-interaction^53^) that in turn affects the equilibrium position of the magnetic ions based on their alignment.^51,52^ A unified, potentially even microscopic model that explains the temperature and field dependent magnetostriction for the entire class of heavy rare earth elements does not exist.^51,52^

### C. Grüneisen concept

In the discussion of the time-resolved strain, we employ a macroscopic, thermodynamic approach that approximates the laser-generated stresses to be directly proportional to the energy densities deposited in the corresponding subsystems. We introduce the main idea at first for the static thermal expansion of Dy. The origin of our approach dates back to 1912 when Grüneisen recognized that the contributions of the lattice vibrations to the volumetric thermal expansion coefficient β(T) of elemental solids and their heat capacity $C_{V}(T)$ at constant volume *V* share the same temperature dependence, so that their ratio can be simplified to a dimensionless, nearly temperature-independent parameter.^54^ The concept of this Grüneisen constant Γ has been employed continuously and was further generalized for the discussion of the thermal expansion in solids.^55–57^ The thermodynamic derivation yields the macroscopic Γ as^55^

$$\Gamma =KV\frac{\beta (T)}{C_{V}(T)},$$ (1)

wherein *K* represents the bulk modulus. This approach can be extended to account for different excitations that contribute energy reservoirs r in a solid by introducing dedicated $\Gamma_{r}$.^55^ The generalization to the case of anisotropic expansion requires the use of anisotropic linear thermal expansion coefficients $\alpha_{i}(T)$ and anisotropic Grüneisen constants Γ_*i*_ as well as the proper directional elastic constants *c_ij_* (Ref. 55) as exemplified for the rare earth Holmium.^58^ For simplicity, we consider the elastic strain response to be purely one-dimensional. This is justified if the probed region is homogeneously excited along the lateral dimension, so that its picosecond response shows no in-plane strain. We thus limit the discussion to the out-of-plane response of the materials so that we will drop the directional indices for the out-of-plane stress $\sigma_{3}=c_{33}\eta_{3}$ in the following. For sufficiently small ΔT, Eq. (1) can be transformed to the linear relation

$$\sigma_{r}=\Gamma_{r}\rho_{r}^{Q}$$ (2)

between the stress *σ_r_* and the laser-induced energy density $\rho_{r}^{Q}(\Delta T)=\int_{T}^{T+\Delta T}C_{r}(T\prime )dT\prime$, wherein the subscript, r, denotes one of the energy reservoirs. For the case of Dy, we separate the total strain response to stress contributions from electronic excitations (r=el), phonons (r=pho), and magnetic excitations (r=mag).

In Fig. 2, we demonstrate the separation of the subsystem contributions to the equilibrium lattice strain and heat capacity in Dy from which we subsequently extract the ratio of the Grüneisen parameters for the combined electron-phonon and magnetic excitations. Using the heat capacity of the chemically equivalent non-magnetic heavy rare earth Lutetium, scaled according to the Debye temperature of Dy, provides an estimate for the combined electron and phonon contribution to the specific heat.^59^ This is indicated by the red shading in Fig. 2(b). The estimated electronic contribution to the heat capacity *C* corresponds to the very small gray shaded area in Fig. 2(b), which we obtain from a Sommerfeld model [$C_{\text{el}}=\gamma_{\text{Dy}}T$ with $\gamma_{\text{Dy}}=4.9$ mJ/(mol K)].^60^ Electronic excitations thus only store a sizeable energy fraction at high electron temperatures that are attained only directly after laser-excitation. For that reason, we label the combined electron-phonon subsystem in the following as phonon contribution (r=pho) unless stated otherwise.

Assuming a constant Grüneisen parameter for the phonon contribution, we obtain an estimate for the thermal expansion of non-magnetic Dy ($\eta_{\text{pho}}$) that we represent by the red line in Fig. 2(a). By subtracting $\eta_{\text{pho}}$ and $C_{\text{pho}}$ from the measured lattice strain and the combined heat capacity, we obtain the contribution of magnetic excitations to the strain and heat capacity, which are indicated by the blue line in Fig. 2(a) and blue shaded area in Fig. 2(b), respectively. From this separation, we can directly extract the strain per deposited energy for the phonon and magnetic subsystem, which is displayed in the inset in Fig. 2(c). Indeed, the linear slope of the magnetic strain in Fig. 2(c) reconfirms the linear relation of stress and energy density of Eq. (2) for the spin system. The linear strain-energy-density relation for the phonon strain is in agreement with previous analysis of the thermal expansion of solids^55,61^ and in particular, the non-magnetic rare earth Lutetium^62^ where Grüneisen parameters Γ are found that are nearly constant over an extended range of temperatures even when *C*(*T*) and β(T) are *T*-dependent. Recently, this was extended to the separated magnetic and nonmagnetic contributions of the thermal expansion of Dy.^32,33^ The slope of the resulting curves is proportional to the Grüneisen parameter since the relevant elastic constant *c*_33_ changes by less than 10% across the displayed temperature region.^63^ The ratio of the Grüneisen constants $\Gamma_{\text{mag}}/\Gamma_{\text{pho}}\approx -3$ indicates that magnetic excitations in Dy are three times more efficient in the stress generation per deposited energy as compared to phonons.

As opposed to the AFM-PM transition that is of second order, the FM-AFM transition is a first order phase transition with a latent heat of 50.7 J/mol.^44^ In our thin film sample, we see that this phase transition occurs between 60 K and 75 K upon heating. We attribute the shift and broadening of the phase transition to epitaxial strains. The FM-AFM phase transition leads to orthorhombic in-plane distortions that are clamped near the interfaces. Since a measurement of the temperature-dependent heat capacity of the Dy transducer within our heterostructure is not possible, we can only specify that the observed negative strain per deposited energy of the spin system is potentially even larger in the region of the FM-AFM transition. Note, however, that this contraction results mainly from the FM-AFM phase transition, since within the FM phase the lattice expands for rising temperatures.

The presented subsystem separation can be applied to gadolinium^30^ and holmium,^31^ which exhibit similarly large negative Grüneisen parameters for magnetic excitations. Heavy rare earth metals are an interesting class of materials for lattice dynamics since their magnetic heat capacity $C_{\text{mag}}$ and the associated entropy of magnetic excitations $\delta S_{\text{mag}}=\Delta Q_{\text{mag}}/T$ are comparable to the phonon contribution over a large temperature region.^31,55,58^ This renders Dy a suitable candidate for experiments that investigate the magnetic contributions, that lead to the NTE response, within a time-resolved experiment.

## III. TIME-RESOLVED EXPERIMENTS

**FIG. 3.**
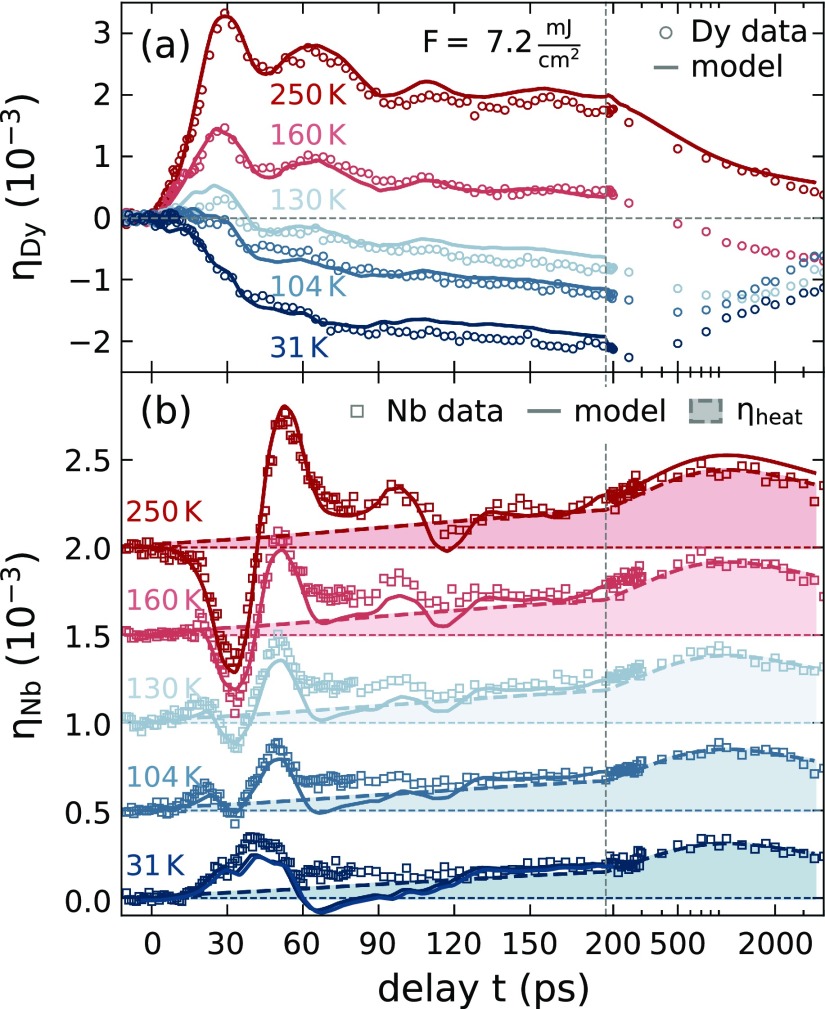
Temperature-dependent strain response of (a) the Dy transducer and (b) the Nb detection layer for a fixed excitation energy density of *F *=* *7.2 mJ/cm^2^. Open symbols represent the average strain of the layers extracted from the Bragg peak shift observed by UXRD. Solid lines represent the simulated UXRD response obtained from a one-dimensional elastic model subjected to time-dependent phonon and magnetic stresses that are detailed in the modeling Sec. IV. The shaded region in (b) indicates the estimated thermal contribution to the Nb strain. The linear to logarithmic axis break (vertical, dashed line) allows the simultaneous comparison of the picosecond strain pulse and the subsequent thermal expansion. Nb curves are offset for clarity.

**FIG. 4.**
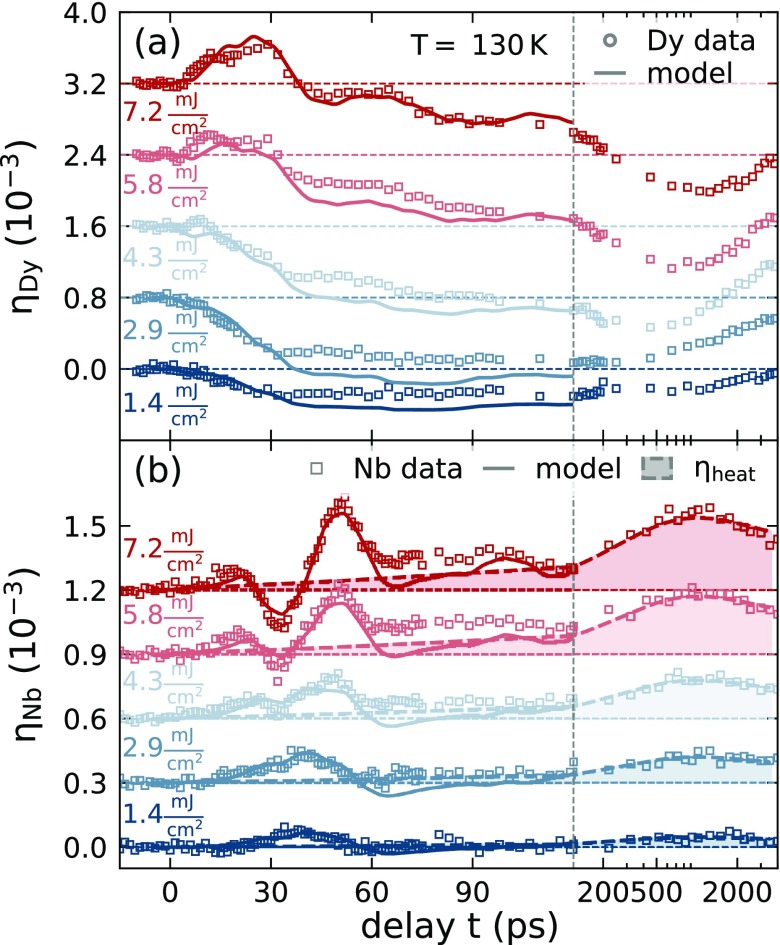
Same depiction as in Fig. 3 used for the excitation energy density dependent strain response of (a) the Dy transducer and (b) the Nb detection layer for the fixed *T *=* *130 K. All curves are offset for clarity. The initial transducer response changes from contraction to expansion as the excitation energy is increased, which coincides with the appearance of the delayed bipolar strain feature in the coherent phonon response of the buried detection layer.

The main experimental results of our study are summarized in Figs. 3 and 4, which display representative picosecond strain responses for both the Dy transducer and the buried Nb detection layer at different starting temperatures *T* and excitation energy densities *F*. In our UXRD experiments, the sample is subjected to 110 fs-long, *p*-polarized laser pulses with a central wavelength of 800 nm at a repetition rate of 1 kHz. The laser excitation profile corresponds to a 2D-Gaussian contour with approximately $1.6\times 1.2\;{\text{mm}}^{2}$ full width at half maximum along its principal axis. The optical pump-pulses are incident under $36^{{}^{\circ}}$ for measuring the Dy response and $40^{{}^{\circ}}$ for the Nb measurements where the angle is given relative to the surface plane of the sample. The x-ray probe-pulses are generated using a laser-based plasma x-ray source,^64^ monochromatized to Cu-K_*α*_-radiation, and focused onto the sample using a Montel optic^65^ with a diamond shaped $300\times 300\;\mu m^{2}$ beam focus on the sample. The sample temperature is monitored via a thermocouple adjacent to the sample and all reported fluence values are provided as incident energy density that is calculated from the incident laser power and the beam footprint.

### A. Temperature dependent UXRD experiments

First, we discuss the results displayed in Fig. 3, where a fixed energy density of 7.2 mJ/cm^2^ is used to excite the sample for different initial temperatures, sampling the different magnetic orders. The results obtained in the PM phase at *T *=* *250 K are displayed by red open symbols and represent the non-magnetic response of the investigated metallic heterostructure. We find an expansion of the Dy layer that reaches its maximum within 30 ps, which corresponds to the time it takes to propagate strain from the air/Y interface through the Dy layer to the Dy/Y interface. After traversing the 5 nm Y interlayer, this expansion enters the Nb layer at approximately 31.5 ps. This expansion pulse is preceded by a compression that results from the fast rise of the spatially inhomogeneous expansive stress. The resulting bipolar shape of the propagating strain pulse is well known in picosecond acoustics; however, optical detection schemes often probe the wave returning back to the surface after a reflection.^1,2,5^

UXRD experiments can monitor the bipolar strain pulse traversing a buried detection layer: The classical bipolar wave first leads to a negative average strain in this layer and before it turns positive, a zero average strain indicates equal expansive and contractive parts in this layer.^35,66^ The nearly bipolar shape of the Nb strain in Fig. 3(b) within the first 75 ps is characteristic of a symmetric strain pulse that is generated by a total stress that rises fast compared to the strain-propagation time through the laser-excited transducer. Echos of the laser-generated picosecond strain pulses that occur due to the partial reflection at the layer interfaces lead to a damped oscillation of the average strain in the Dy transducer and a second bipolar feature in the Nb detection layer between 80 ps and 125 ps.

In addition to these signatures of the coherent phonon wave packets, we observe a slowly increasing strain that originates from the excitation of incoherent phonons in the materials. This thermal expansion contribution of the excited Dy transducer is observed to decay on a nanosecond timescale by heat diffusion toward the substrate. This diffusion leads to a transient thermal expansion in the Nb detection layer that exhibits its maximum at approximately 1 ns after the excitation. To highlight the contribution of heat transport to the Nb expansion, we added dashed lines and shaded areas to Fig. 3(b). These lines are all scaled copies $A(T){\bar{\eta }}_{\text{heat}}(t)$ of the transient average strain ${\bar{\eta }}_{\text{heat}}(t)$ in the Nb layer, which fit the 250 K data. This line could be drawn through the data by averaging out any coherent oscillations, but here we used the simulated expansion according to a Fourier heat law model discussed below. The temperature dependent factor *A*(*T*) is determined by scaling the temperature-independent ${\bar{\eta }}_{\text{heat}}(t)$ to the Nb data between 200 ps and 3 ns. *A*(*T*) decreases significantly with temperature *T*, indicating that part of the energy density is stored in magnetic excitations that become accessible below $T_{Né\text{el}}$ within the Dy transducer. The details of the modeling including a plot of *A*(*T*) are discussed in Sec. IV.

Looking at the short timescale, we find that the magnetic excitations accessible upon lowering the temperature transform the transducer response continuously from a rapid expansion to a relatively slow contraction [Fig. 3(a)]. The coherent phonon oscillations in Dy become significantly weaker. In the Nb-detection layer [Fig. 3(b)], this coherent picosecond strain wave is observed to change from a large bipolar shape with a leading compression to a weaker unipolar expansion. At intermediate temperatures, a bipolar strain wave with reduced amplitude compared to the PM phase is preceeded by an expansion, which we attribute to contractive stress at the back side of the Dy layer, which absorbs the smallest energy density.

### B. Excitation density dependent strain-response

This hypothesis was checked by the excitation-density dependent measurements that we depict in Fig. 4. We repeated the UXRD experiment for a systematic variation of excitation fluences at a fixed initial sample temperature of *T *=* *130 K in the AFM phase of Dy. The increase in the deposited energy in the heterostructure can be seen directly as an increase in the transient thermal expansion of the Nb detection layer beyond 100 ps. The shaded area in Fig. 4(b) again indicates our estimate of the incoherent strain contribution that we obtain by scaling the strain ${\bar{\eta }}_{\text{heat}}(t)$ from the PM phase to approximate the data between 0.2 and 3 ns. The resulting amplitude A(130 K,F) scales super-linearly with *F*, indicating a saturation of the energy transfer to magnetic excitations (cf. Sec. IV E). Note that the magnetic contribution to the specific heat $C_{\text{mag}}$ above $T_{Né\text{el}}$ is small but finite [Fig. 2(b)]. According to the equilibrium analysis, the Grüneisen constant $\Gamma_{\text{mag}}$ has the same value above $T_{Né\text{el}}$, as the slope in Fig. 2(c) remains constant toward the end. Similar to the temperature dependent experiments, we find a strong qualitative change of the picosecond strain response in our heterostructure. For low excitation energies *F *<* *4.3 mJ/cm^2^, we observe an average contraction of the Dy transducer that goes along with the unipolar expansion of the Nb layer. For *F *>* *4.3 mJ/cm^2^, we observe an initial expansion of the Dy transducer that changes to an average contraction at delays much larger than the 30 ps that it takes for strain propagation through the layer.

The strain pulse measured in the Nb detection layer [Fig. 4(b)] exhibits a unipolar expansion feature for low excitation energy densities. For higher fluences, this expansion is superimposed by a bipolar strain response expected for the excitation of phonons, i.e., the dominant signal in the PM phase. However, here we confirm that the bipolar strain pulse is preceded by an expansion. Since this leading expansion is rationalized by a contractive stress at the backside of the Dy transducer, the high fluence data directly show that the magnetic excitations at the back side of the Dy transducer are not saturated. The simultaneous expansion at the Dy front side, however, triggers the bipolar waveform which is observed in the detection layer with a delay given by the sound propagation. This waveform confirms a saturation of the magnetic excitation in the Dy front part, because it is the trailing part of the strain pulse, which is considerably changed by the increasing fluence. The fraction of the transducer, where expansive stress from the combined electron-phonon system dominates over the contractive stress, extends over a thicker part of the layer for higher excitation energy densities.

Before discussing the resulting quantitative findings and the modeling approach, we briefly summarize the experimental conclusions that can be drawn directly from the presented UXRD data. For low excitation energies and temperatures, we observe that the magnetic rare earth transducer contracts on average as opposed to the expansion that is observed in the PM phase. For intermediate temperatures, we can infer that the front of the transducer expands while its backside contracts. This behavior can be rationalized by a spatially dependent saturation of the magnetic stress that originates from the inhomogeneous energy deposition profile. We find that the strain propagation maps the stress profile onto a nearly background-free, time-dependent signal of the average strain in the buried Nb detection layer. Since heat diffusion takes longer than strain waves, detecting the propagated strain wave in the detection layer separates the strain contribution of coherent and incoherent phonons, which are superimposed within the photoexcited Y and Dy layer. The additional layer thus provides a nanometric depth-resolution of the UXRD experiments using hard x-rays, which otherwise exhibit an extinction length on the order of few *μ*m. The heat transport observed via the thermal expansion of the adjacent Nb layer is found to be reduced below the magnetic ordering temperatures. This can be directly seen in the area under the curve for the average strain in Nb [Fig. 4(b)], which is smaller at low temperatures. This is quantitatively depicted in Fig. S1(c) of the supplementary material.

## IV. MODELING SPATIO-TEMPORAL STRESS

Our modeling approach is designed to identify the ingredients that are necessary to rationalize the observed temperature- and excitation fluence-dependent strain-response of the magnetic rare earth transducer. To that end, we model a time- and space dependent driving stress $\sigma_{\text{tot}}(z,t)=\sigma_{\text{pho}}(z,t)+\sigma_{\text{mag}}(z,t)$ generated by energy transfer to magnetic excitations that acts in addition to the electron-phonon stress calibrated by the response in the PM phase. The measured average strain $\bar{\eta }$ for *t *>* *100 ps only encodes the average stress in the Nb layer originating from the heat diffusion. The qualitative analysis of the strain wave detected in Nb for *t *<* *100 ps already indicated that we are able to extract temporal variations σ(z,t) of the spatial stress profile in the Dy by modeling the strain wave launched into the Nb detection layer. As shown in Figs. 3 and 4, we find qualitative and quantitative agreement between our model and data.

In the following, we first discuss the general assumptions of our modeling approach before we specify the simulation steps and assumptions for the modeled electron-phonon stress and the magnetic stress contributions.

### A. General model assumptions

**FIG. 5.**
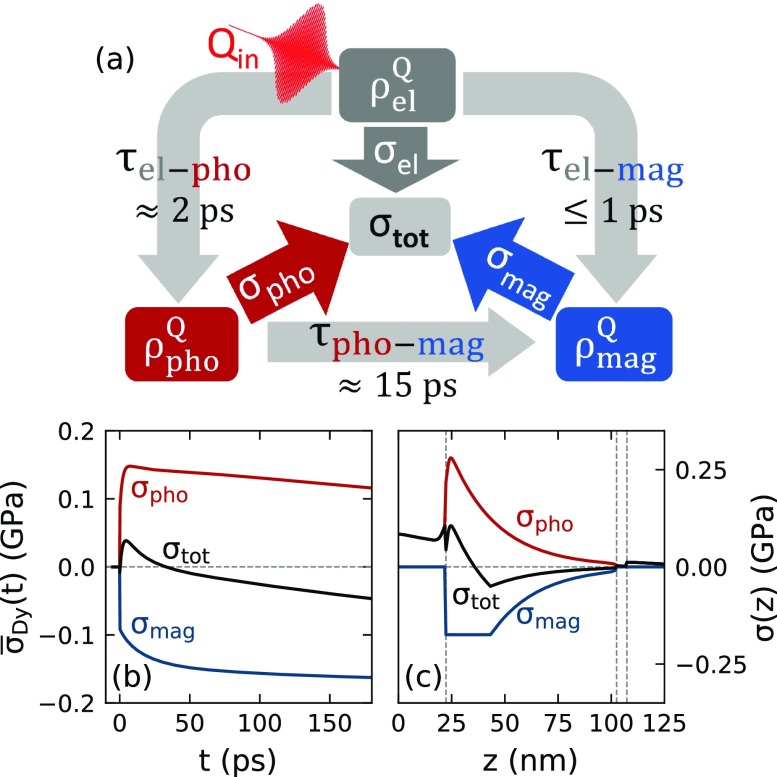
(a) Schematic stress contributions used in the modeling approach. Arrows and labels indicate the modeled energy transfer processes between the subsystems and the assumed coupling-timescales *τ*. The resulting time-dependence of the spatially averaged stress contributions in Dy (b) and the used stress profile 100 fs after excitation (c) are shown exemplarily for *T *=* *130 K and *F *=* *7.2 mJ/cm^2^. Vertical dashed lines in (c) indicate interfaces of the Y/Dy/Y/Nb heterostructure. This illustrates that the total stress evolution is a superposition of the expansive electron-phonon stress and the contractive magnetic stress both in the space- and time-domain.

We assume the energy transfer processes between the stress-contributing subsystems schematically shown in Fig. 5(a), with coupling-times that correspond to the stress rise-times depicted in Fig. 5(b). The spatial stress profiles in the Dy layer are exemplified in Fig. 5(c) for the parameters *T *=* *130 K and *F *=* *7.2 mJ/cm^2^ that are representative of the AFM sample response. The total energy provided by the laser pulse is distributed between electron-phonon excitations that exert an expansive stress and magnetic-excitations that exert a contractive stress within the Dy layer. The energy is assumed to be deposited with the same inhomogeneous spatial profile to phonon- and magnetic excitations. The magnetic excitations however induce a contractive stress whose amplitude is three times larger than the expansive stress that is generated by electron-phonon excitations with the same energy density, according to the static Grüneisen analysis [Fig. 2(c)]. We keep as many simulation parameters as possible constant throughout the modeling. This includes the three coupling times illustrated in Fig. 5, the ratio of the initial energy redistribution from electrons to spins and phonons, and the maximum value for the spin energy density given by the saturation value $\rho_{\text{mag}}^{\text{sat}}$. We assume that the spatial energy distribution in the magnetic subsystem follows the profile of the phonon energy-density obtained by modeling the PM phase. This profile changes in time according to the heat diffusion, and we implicitly assume that the coupling of energy from phonons to spins is effective enough to prevent different spatial profiles of the contributing excitations. However, when the magnetic energy density exceeds the saturation value $\rho_{\text{mag}}^{\text{sat}}$, we truncate it. This truncation yields the non-monotonous spatial variation of the total driving stress as a function of temperature and excitation fluence. The temporal variation owes to the fact that the fraction of energy coupled rapidly to the phonon system is larger than into the magnetic system. Therefore, the additional 15 ps spin-phonon coupling time leads to a slow rise of the spin excitation and a decay of the phonon excitation.

We limit the modeling time to 180 ps since we expect remagnetization effects to significantly contribute to the strain at later times via magnetostriction. A full description would require a detailed model for the recovery of the magnetic order including both thermal transport and nucleation, growth, and coalescence of magnetic domains. This is beyond the scope of our one-dimensional thermodynamical approach. We furthermore refrain from a time- and space-dependent three-temperature model that has been previously used to rationalize the demagnetization of the magnetic specimen,^67,68^ because it would require many, potentially temperature dependent, material constants for all layers in our heterostructure that are not known with the required accuracy. To what extend a three temperature model would capture the demagnetization and remagnetization of both the itinerant (5*d*6*s*) conduction band electrons and the localized 4*f* electrons in heavy rare earth metals is a matter of current research debate.^39,69,70^

### B. Simulated spatio-temporal stress contributions

**FIG. 6.**
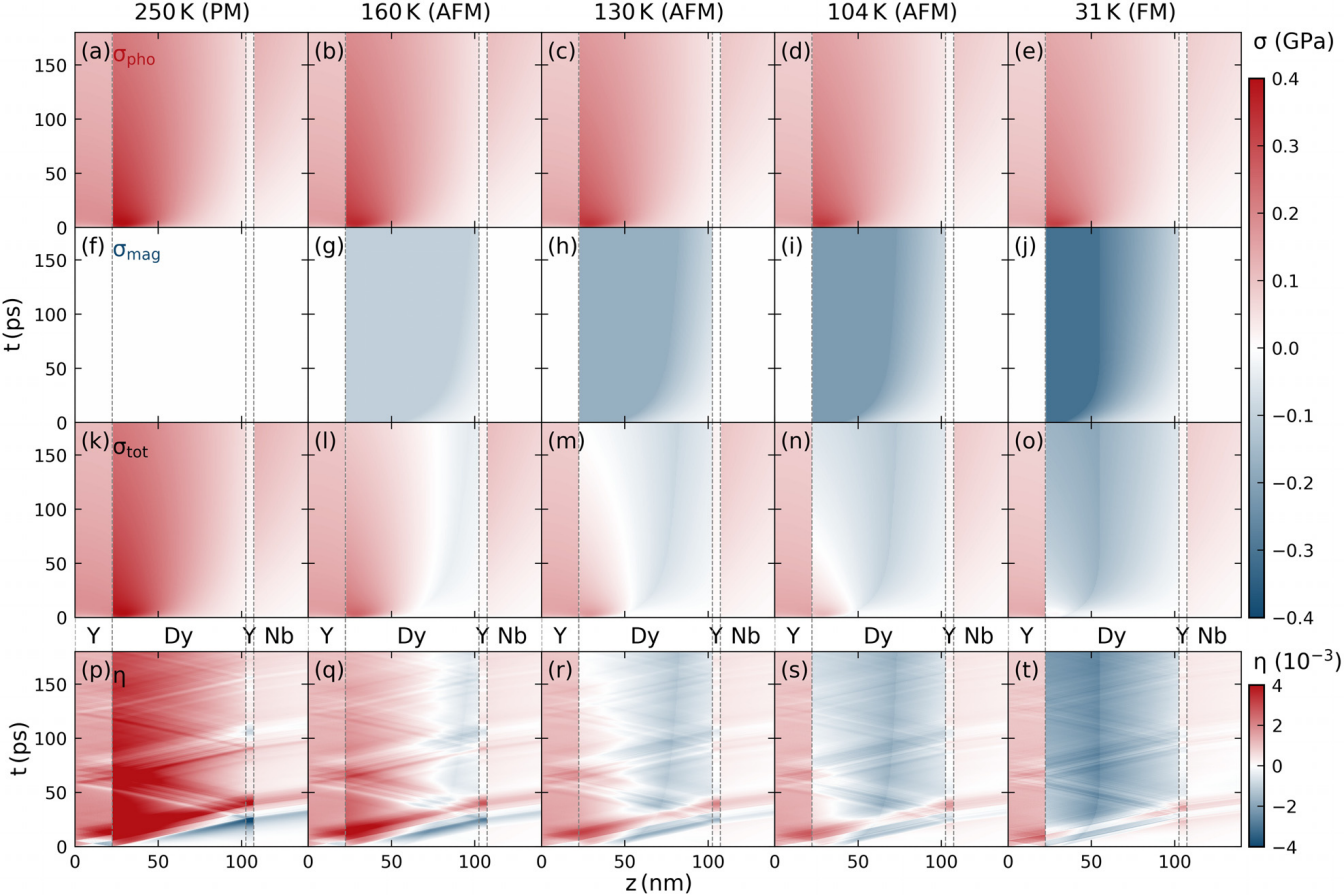
Modeled spatiotemporal stress and strain response in our heterostructure for a fixed excitation with *F *=* *7.2 mJ/cm^2^ at different starting temperatures. The phonon-stress contributions (a)–(e) and the magnetic-stress contributions (f)–(j) in the first and second row add to the total stress provided in the third row (k)–(o). This illustrates the temperature and time-dependent energy transfer and the saturation effects due to the finite amount of energy that can be transferred to magnetic excitations. The resulting strain response of a linear chain of masses and springs model that contains contributions from both coherent and incoherent phonons is shown at the bottom (p)–(t). These strain maps are used to simulate the time-dependent Bragg peak shift that yields the modeled Dy and Nb strain response in Fig. 3. Vertical dashed lines indicate the interfaces within the Y/Dy/Y/Nb heterostructure.

The modeled time-dependent stress profiles, separated into the expansive, contractive and the total stress contributions are displayed in Figs. 6(a)–6(o). The first row [Figs. 6(a)–6(e)] shows the expansive electron-phonon stress ($\sigma_{\text{pho}}$). In the PM-phase (250 K), $\sigma_{\text{pho}}$ is identical to the total stress ($\sigma_{\text{tot}}$), shown in the third row. For the low temperature magnetic phases, the energy transfer to magnetic excitations reduces the amplitude of $\sigma_{\text{pho}}$ [Figs. 6(b)–6(e)] without changing the relative spatio-temporal form, and at the same time, it creates the magnetic-stress contribution ($\sigma_{\text{mag}}$) shown in the second row [Figs. 6(f)–6(j)]. The resulting total stress $\sigma_{\text{tot}}$ [Figs. 6(k)–6(o)] strongly depends on the initial temperature and excitation energy. This is supported by the modeled spatiotemporal stress profile and strain response for the excitation energy density dependent experiments at *T *=* *130 K that we display in Sec. S5 of the supplementary material. The modeled strain response [Figs. 6(p)–6(t)] contains the propagating strain pulses that are launched at gradients of the driving stress in addition to the strain contribution of the local driving stress.

While the saturation level of the magnetic stress changes for the *T*-dependent measurements, *F*-dependent measurements vary the deposited energy density for a fixed maximum magnetic stress. In both cases, we observe that the absolute value of the total stress that acts on the lattice is reduced in comparison to the competing contributions from magnetic- and phonon excitations. Even though the total stress is contractive at the end of most simulation scenarios, the maximum contraction is attained slower than the magnetic stress rise time due to the time-dependent balance of phonon and magnetic stresses. The reappearance of the bipolar strain pulse in the detection layer response for high excitation energies thus occurs since the total stress at the top Y/Dy interface is dominated by an unbalanced electron-phonon stress.

### C. Simulation steps

We use the modular udkm1Dsim^71^ MATLAB library to model the time-dependent energy density, strain, and x-ray reflectivity of the non-magnetic heterostructure response. The solid, dark-red line in Figs. 3(a) and 3(b) corresponds to the strain that we obtain from the simulated transient x-ray diffraction peak shift. In the first step, we calculate the time-dependent temperature changes upon laser excitation using a one-dimensional Fourier heat diffusion model from the thermophysical properties of the materials in the PM phase that we list in Sec. S3 of the supplementary material. The resulting spatiotemporal energy density is subsequently translated to a stress that acts as the driving force on a linear chain of masses and springs, which calculates the time-resolved layer strain with unit-cell resolution. The final simulation step employs a transfer matrix algorithm that yields the Bragg-peak evolution of the strained sample according to dynamical x-ray diffraction theory. Section S3 of the supplementary material shows a flow chart of the simulation steps including the relevant equations. The good quantitative agreement between UXRD data and simulation indicates that our model is a suitable representation of the sample properties and non-magnetic processes (i.e., layer thicknesses, optical excitation parameters, stress profiles, and rise-times) in the PM phase of Dy. We keep all the parameters given in Table I of the supplementary material fixed throughout the modeling, even in the FM and AFM phase, since these parameters describe the electron-phonon system. Section IV E describes how we added the contractive negative stress according to Eq. (2) for excitation in the AFM and FM phase.

### D. Electron-phonon stress contribution

Both the Dy and the Nb strain responses in the PM-phase are used to calibrate the electron-phonon stress contribution. The electron-phonon coupling is included by a 2 ps time-constant of the expansive stress and a ratio between the Grüneisen constants $\Gamma_{\text{el}}/\Gamma_{\text{pho}}=$ 0.5 for both the Y and Dy layers. These parameters are chosen to fit the observed expansion of the Dy layer and the coherent phonon oscillation amplitude of the PM response and are fixed for the subsequent modeling. To illustrate the effect of the stress rise time on the shape of the strain pulse seen by UXRD in the detection layer, we present the elastic-response-simulations of the average strain within a simplified structure that contains only a transducer and a detection layer in Sec. S2 of the supplementary material. To recover the observed direct rise of the Dy strain after laser excitation, we have to assume a reduced absorption and thermal expansion in the 21 nm thick Y capping layer, which would otherwise compress the adjacent Dy. Although the estimated expansion of the Y layer is reduced by 40% compared to the literature value, we stress that this parameter is kept fixed for all subsequent simulations that focus on the *T*-dependent magnetic response. One possible explanation for this deviation could be a partial oxidation of the capping layer since the metallic sample was kept at ambient conditions prior to the measurements.

To match the slow nanosecond decay of the Dy strain as well as the delayed rise of the expansion in the Nb layer, we use an effective thermal conductivity value for the Dy layer that is reduced by approximately 40% compared to the bulk literature value to account for thermal interface resistance effects. Using the material-specific elastic constants, we can then transform the energy densities $\rho_{r}$ into an estimated spatiotemporal profile of the driving stress $\sigma_{r}=\Gamma_{r}\rho_{r}$ according to the Grüneisen concept [Figs. 6(a) and 6(k)]. This stress drives the elastic response shown in Fig. 6(p). The modeled strain matches both the Dy transducer and Nb detection layer response qualitatively and quantitatively over the entire 3.5 ns simulation time as shown in Fig. 3.

### E. Magnetic stress contribution

**FIG. 7.**
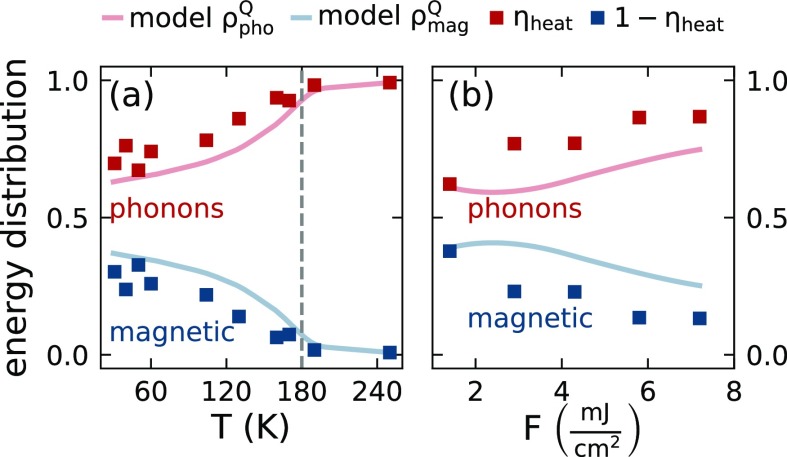
Estimated energy distribution between magnetic- and phonon excitations within the rare earth transducer for the presented temperature- (a) and excitation energy dependent (b) measurements at fixed *F *=* *7.2 mJ/cm^2^ and *T *=* *130 K, respectively. Data points correspond to the fitted amplitude *A*(*T*, *F*) of the thermal expansion $A(T,F)\eta_{\text{heat}}(t)$ of the Nb detection layer (shaded areas in Figs. 3 and 4), normalized to the PM phase value A(250 K). Solid lines represent the energy distribution at the last time step *t *=* *180 ps of the spatiotemporal stress–strain simulation. The fraction of deposited energy in the spin system decreases when approaching $T_{Né\text{el}}$ from low temperatures as well as for higher fluences.

To minimize the number of parameters for the simulations in the AFM and FM phase, we not only keep all thermophysical parameters describing the e-ph system fixed. We also use the shape of the spatiotemporal phonon stress-profile $\eta_{\text{heat}}(z,t)$ extracted in the PM phase. We reduce its amplitude in all layers (Y, Dy and Nb) according to the fraction of the energy that is transferred to magnetic excitations that is shown in Fig. 7 as red squares. The blue squares represent $1-\eta_{\text{heat}}$, which is proportional to the fraction of energy that is stored in the magnetic system of Dy on the few ns timescale. The lines in Fig. 7 show the relative weight of the energy densities in the phonon (red) and magnetic system (blue) in our model. We find a qualitative agreement with the data if locally a fraction of 25% of the excitation energy is deposited instantaneously into the magnetic system to account for subpicosecond electron-spin couplings and additionally a second energy fraction of 25% of the excitation energy is subsequently transferred from phonon to magnetic excitations by the phonon-spin coupling. Only when this energy transfer locally exceeds the maximum energy density $\rho_{\text{mag}}^{\text{sat}}$ in the spin system, we truncate the energy transfer from phonons to spins. Thus, to account for the experimentally observed reduced energy transport to the adjacent layer in the magnetic phase of Dy, we reduce the energy density in the Nb and Y layers and add the removed fraction to the Dy layer where the energy is distributed between magnetic and phonon excitation. We use $\rho_{\text{mag}}^{\text{sat}}$ as a free parameter in the modeling and find the best agreement with our data with $\rho_{\text{mag}}^{\text{sat}}(T_{\text{start}})=0.81\int_{T_{\text{start}}}^{\infty }C_{\text{mag}}(T\prime )dT\prime$, using the literature bulk values for $C_{\text{mag}}(T\prime )$. We believe that either the thin film value for $C_{\text{mag}}(T\prime )$ is smaller compared to the bulk value, or only a reduced fraction of the magnetic heat capacity of the thin film is accessible upon ultrafast laser excitation. Our model reproduces the trends of the experimentally observed reduced energy flow in Fig. 7 as a function of the starting temperature and the fluence correctly. The energy fraction that is not detected in Nb persists in the magnetic excitations of Dy that act as a saturable heat sink. The saturation can be seen by the reduced energy fraction in the magnetic excitations closer to $T_{Né\text{el}}$ and the decrease in the magnetic energy fraction for higher excitation densities. The blue lines representing the fraction of heat in the magnetic system around 180 ps in our model report a systematically larger value than the blue squares derived from the experiment of the Nb strain between 0.2 and 3 ns. This is in line with the previous analysis of persistent magnetic excitations in Dy decaying within a few ns.^32^

We use energy transfer times that are in agreement with recent ultrafast demagnetization studies in AFM-dysprosium^38^ and holmium.^39^ The resonant diffraction studies independently report both a subpicosecond demagnetization, which is attributed to electron-spin coupling and a second demagnetization timescale on the order of 15 ps, which is attributed to phonon-spin coupling. Systematic modeling of our data yields that both timescales are necessary to capture the early time strain response of the transducer. A substantial fraction of the magnetic stress needs to be present within the first ps to balance the otherwise expansive electron-phonon response. However, our modeling shows that not all energy is instantaneously transferred to magnetic excitations since the resulting stress would drive a contraction that is significantly faster than the observed response.

In addition to these two intrinsic demagnetization timescales, we observe a contraction of the Dy transducer for larger pump-probe delays. This effect is most pronounced at high excitation densities and *T* close to and above $T_{Né\text{el}}$. Our simulations show that this can be rationalized by two energy transport effects within the inhomogeneously excited heterostructure. First there is thermal diffusion of phonon excitations within the inhomogeneously heated Dy. This transports energy from the near surface region where spin-excitations are saturated into the depth of the transducer, where the spin energy density is below the saturation threshold. The coupling of energy from phonons to the spins decreases the expansive phonon stress and increases the contractive magnetic stress contribution. The transport of phonon heat out of the Dy layer into the Nb reduces the expansion further. This allows us to model the spatial spin-stress profile. Although the quantitative link between the magnetic stress and the sublattice magnetization in the time-domain is largely unexplored, we can argue qualitatively that the saturation of the spin-stress will be linked to regions of complete demagnetization. For static thermal expansion experiments, it has been reported that the magnetostrictive stress is proportional to the square of the sublattice-magnetization.^43,72^ Thus, we can speculate that the modeled magnetic stress profile is qualitatively similar to the demagnetization profile. More precisely, we assume that the observed magnetic stress profile reflects the demagnetization of the 4*f*-electrons of the heavy rare earth. These anisotropic orbitals carry large, localized magnetic moments that distinguish rare earth materials from 3*d*-transition metals that exhibit by far smaller magnetostriction.

The color code of Fig. 6 provides a qualitative overview over the temperature dependent stress contributions that we focus on in the current report. For a more quantitative comparison, we refer the reader to Sec. S5 of the supplementary material. There we show outlines of the modeled spatial stress profiles at 6 ps, 45 ps, and 180 ps alongside the time dependence of the modeled stress contributions for both the *T*- and *F*-dependent experiments.

Our current modeling provides a plausible scenario for the driving stress. A satisfactory agreement between the data and modeling definitively requires three energy transfer timescales to the spin system. The first energy transfer to the magnetic excitations has to rise equally fast or faster than the electron-phonon stress to cancel the expansive electron-phonon stress in the first few ps. The second timescale has to be in the range of 10–20 ps to model the contraction of the Dy layer and the unipolar expansion wave observed in the Nb layer. The third process on a timescale larger than 40 ps is given by thermal transport within the Dy layer. This is the simplest possible temporal behavior of the stress that is consistent with our data.

So far, we have also treated the FM and AFM response equally although there exists an additional first order phase-transition in the low temperature phase and the demagnetization of the ferromagnetic phase was reported to occur slower than in the antiferromagnetic Dy.^38^ We attribute the reasonable agreement for the FM phase at *T *=* *31 K to the use of a large excitation energy density that drives mainly spin excitations with the calibrated Grüneisen constant of the AFM phase. These magnetic-excitations seem to dominate the magnetostrictive response at high excitation energy densities compared to the potential contribution of the latent heat that is necessary to undergo the metamagnetic FM-AFM transition. A detailed study of the magnetostriction at the FM-AFM transition is deferred to future investigations.

## V. CONCLUSION

The presented picosecond strain dynamics in a laser-excited heterostructure containing a rare-earth transducer shows strong magnetic contributions to the lattice response. Both the picosecond strain pulse and the thermal transport are affected by energy transfer processes to magnetic excitations. The transient strain observed in a buried detection layer directly shows the saturation of the contractive magnetic stress component, which occurs when an increasing fraction of the Dy layer is excited across its magnetic phase transition. The spatially varying sign of the stress within the Dy layer triggers unconventional strain pulses, which exhibit a leading expansive part in front of the conventional bipolar strain pulse. Our modeling yields an estimate for the time- and space-dependent profile of the additional magnetic stress contribution to the lattice dynamics. The magnetic excitations act as a saturable heat reservoir, which stores a significant fraction of the excitation energy and exerts a contractive stress that dominates over the phonon contribution. We expect this finding to be generic for the magnetic phase in the class of heavy rare earth materials in the periodic table of elements ${}_{64}\text{Gd}$–${}_{69}\text{Tm}$. The observed energy transfer timescales are in agreement with recent demagnetization experiments. This indicates that the magnetostrictive response can be used to probe the time-dependent evolution of the sublattice magnetization.

Our investigation demonstrates the capabilities of UXRD experiments in a transducer-detector geometry to observe non-trivial spatial stress profiles. We emphasize that extracting the stress profile in the transducer by UXRD from an adjacent crystalline detection layer can be applied to investigate non-crystalline transducer films. The combination of picosecond acoustics experiment, UXRD detection, and elastic modeling can be used to study the strain generation by energy transfer to other degrees of freedom. This comprises, in particular, phase-transition effects such as studies of ferroelectric and charge order transition as well as investigations of other magnetostrictive materials, which may hold hitherto unknown functionalities.

## SUPPLEMENTARY MATERIAL

See the supplementary material for a complete overview of the UXRD results (Sec. S1) and simulations (Sec. S2) that illustrate the effect of the stress rise time, stress profile length, and the detection layer thickness for the strain response in a simplified transducer-detector heterostructure. Section S3 provides an overview of the mathematical relations relevant for the strain simulation and a list of the relevant thermophysical material properties, including a description of the Poisson correction factor for the Grüneisen constant. Section S4 shows a detailed plot of the spatiotemporal stress contributions for the *T*- and *F*-dependent modeling that comprises stress-profiles at selected times and the time dependent average stress within the Dy layer.
